# Prevalence and outcomes of chronic comorbid conditions in patients with sepsis in Korea: a nationwide cohort study from 2011 to 2016

**DOI:** 10.1186/s12879-024-09081-x

**Published:** 2024-02-12

**Authors:** Christine Kang, Seongmi Choi, Eun Jin Jang, Somin Joo, Jae Hoon Jeong, Seung-Young Oh, Ho Geol Ryu, Hannah Lee

**Affiliations:** 1 Departments of Critical Care Medicine, Seoul National University Hospital, Seoul, South Korea; 2 Korea Real Estate Board, Daegu, South Korea; 3 Department of Information Statistics, Andong National University, Andong, South Korea; 4 Departments of Anesthesiology and Pain Medicine, Seoul National University Hospital, Seoul, South Korea; 5 Departments of Anesthesiology and Pain Medicine, Seoul National University, College of Medicine, Seoul, South Korea

**Keywords:** Sepsis, Hypertension, Diabetes mellitus, Liver cirrhosis, Chronic kidney disease, Malignancy, Mortality

## Abstract

**Background:**

Chronic comorbid conditions are common in patients with sepsis and may affect the outcomes. This study aimed to evaluate the prevalence and outcomes of common comorbidities in patients with sepsis.

**Methods:**

We conducted a nationwide retrospective cohort study. Using data from the National Health Insurance Service of Korea. Adult patients (age ≥ 18 years) who were hospitalized in tertiary or general hospitals with a diagnosis of sepsis between 2011 and 2016 were analyzed. After screening of all International Classification of Diseases 10th revision codes for comorbidities, we identified hypertension, diabetes mellitus (DM), liver cirrhosis (LC), chronic kidney disease (CKD), and malignancy as prevalent comorbidities.

**Results:**

Overall, 373,539 patients diagnosed with sepsis were hospitalized in Korea between 2011 and 2016. Among them, 46.7% had hypertension, 23.6% had DM, 7.4% had LC, 13.7% had CKD, and 30.7% had malignancy. In-hospital mortality rates for patients with hypertension, DM, LC, CKD, and malignancy were 25.5%, 25.2%, 34.5%, 28.0%, and 33.3%, respectively, showing a decreasing trend over time (*P* < 0.001). After adjusting for baseline characteristics, male sex, older age, use of mechanical ventilation, and continuous renal replacement therapy, LC, CKD, and malignancy were significantly associated with in-hospital mortality.

**Conclusions:**

Hypertension is the most prevalent comorbidity in patients with sepsis, and it is associated with an increased survival rate. Additionally, liver cirrhosis, chronic kidney disease, and malignancy result in higher mortality rates than hypertension and DM, and are significant risk factors for in-hospital mortality in patients with sepsis.

**Supplementary Information:**

The online version contains supplementary material available at 10.1186/s12879-024-09081-x.

## Introduction

Sepsis is a systemic and life-threatening host response to infection that can be complicated by severe organ dysfunction and requires significant healthcare resources. Chronic comorbid conditions are clinically common in patients diagnosed with sepsis. According to previous reports, 55.5% to 65% of patients with sepsis had underlying comorbidities [1, 2]. As underlying chronic diseases may impact the outcomes among patients with sepsis, an assessment on the pre-existing comorbidities is important to improve the prediction of mortality from sepsis [3].

Previous epidemiological studies have observed that chronic medical comorbid conditions such as end stage renal disease, malignancy, liver cirrhosis (LC) are associated with an increased risk of developing sepsis and sepsis-related mortality [4–6]. In contrast, there is controversy regarding the impact of comorbidities such as diabetes mellitus (DM) and chronic kidney disease (CKD) on patient outcomes in patients with sepsis [7–10]. However, there are few reports regarding the prevalence and outcomes of several common comorbidities in patients with sepsis by a comprehensive analysis using a nationwide magnitude of epidemiologic data.

Therefore, we aimed to investigate the prevalence and outcomes of common chronic medical comorbidities including chronic hypertension, DM, LC, CKD, and malignancy in patients with sepsis from 2011 to 2016 in Korea. Additionally, we evaluated risk factors for in-hospital mortality and the trends of in-hospital mortality and those comorbidities over time.

## Materials and methods

The institution review board approved this retrospective observational study, waiving informed consent because the study was retrospective in nature.

### Database

Data were obtained from the National Health Insurance Service (NHIS) database, Korea’s national database for reimbursable medical claims. The NHIS database contains all claims data of the population covered by the National Health Insurance (NHI) and Medical Aid programs in Korea. The Medical Aid program covers the lowest-income groups, which constitute 3% of the total population. The National Health Insurance (NHI) Program is administered by the National Health Insurance Service (NHIS), and the covered treatments are reviewed by the Health Insurance Review and Assessment Service (HIRA). NHIS approves medical reimbursement claims for over 97% of the Korean population. It serves every type of healthcare facility, from private practices to general and tertiary hospitals. In our study, we exclusively considered the initial episode of sepsis for each patient, excluding any subsequent occurrences. The claims data includes encrypted personal information such as age, sex, name, resident registration number. It also contains primary and secondary diagnoses, in-hospital deaths, length of stay (LOS), and healthcare costs reimbursed by the HIRA. To calculate the incidence of sepsis, we used the 2011–2016 mid-year population census from the Korea Statistical Information Service, categorized by sex and age, as the denominator.

### Patient selection and study protocol

We identified adult patients (aged ≥18 years) discharged with a diagnosis of sepsis from tertiary or general hospitals in Korea between 2011 and 2016, using International Classification of Diseases, Tenth Revision (ICD-10) codes (see Supplemental Table 1). We identified hypertension with I10-I15, DM with E10-E14, LC with K70.3, 71.7, and 74, CKD with N18, malignancy with C00–97 and D00–09, locoregional solid tumors with C00–78, 80, and D00–09, metastatic cancer with C79, and hematologic malignancy with C81–96. Hospital admissions in the same year as the index date were used to evaluate comorbidities and the Elixhauser comorbidity index. Patients were considered to have no comorbidities if they were not hospitalized in the year before the index date. Continuous renal replacement therapy (CRRT) refers to patients who received CRRT during hospitalization after the sepsis index date and have not previously received intermittent hemodialysis, peritoneal dialysis, or CRRT.

To adjust for disease severity, we used as a covariate the Elixhauser comorbidity index, which is derived from 30 disease entries using ICD-10 codes [11] and has been known to correlate with hospital mortality, [12] was used as a covariable to adjust for severity of illness. In-hospital mortality, ICU length of stay, and hospital length of stay were also extracted.

To calculate the crude sepsis incidence by year, we used the mid-year population count. The adjusted sepsis incidence was calculated and compared according to year, age group, and sex, using the 2011 mid-year population data as a baseline population. The incidence rate ratios of sepsis in patients with the five comorbidities were compared between 2011 and 2013 and 2014–2016, which was based on the introduction of the sepsis guidelines in 2013 [13]. Data regarding LOS and ICU care requirements were also collected and evaluated. Discharge date and resident registration number were used to link patients transferred to other hospitals for further treatment and medical records.

### Statistical analysis

Data were analyzed with SAS 9.2 software (SAS institute Inc., Cary, NC). Incidence of sepsis was calculated after adjusting for age and sex. Comparisons of clinical outcomes between age groups, sex, and year were performed. Chi-square tests were used to compare categorical variables including comorbidities, in-hospital mortality, use of MV (mechanical ventilation) and CRRT, requirement of ICU admission, type of hospital, and insurance state. Categorical variables were presented as frequency (percentage), and continuous variables as mean (standard deviation). The differences in the LOS between calendar years were analyzed using ANOVA test. Poisson regression was used to calculate the incidence rate ratios of sepsis, severe sepsis and septic shock after adjusting mean Elixhauser comorbidity index, age group, sex, and calendar year group. Negative binomial regression model was used to compare the trends of incidence of sepsis, severe sepsis after adjusting age, sex, and Elixhauser comorbidity index. The Cochran-Armitage trend test was used to analyze in-hospital mortality and ICU admission rates for annual trends. And logistic regression analysis was used to identify the risk factors for in-hospital mortality. A *P-value* < 0.05 was considered statistically significant.

## Results

From 2011 to 2016, 373,539 patients were admitted to a general or tertiary hospital with a diagnosis of sepsis. The mean age was 68.9 ± 15.3 years. Patient characteristics are summarized in Table 1.

**Table 1:** Characteristics of patients with sepsis

Characteristics	Total (*N* = 373,539)	2011 (*N* = 49,116)	2012 (*N* = 54,622)	2013 (*N* = 58,092)	2014 (*N* = 60,608)	2015 (*N* = 66,511)	2016 (*N* = 84,590)	*P*
**Sex,** ***n*** **(%)**	373,539 (100)	49,116(100)	54,622 (100)	58,092 (100)	60,608 (100)	66,511 (100)	84,590 (100)	
**Male**	186,833 (50.0)	25,362 (51.6)	28,128 (51.5)	29,414 (50.6)	30,220 (49.9)	32,502 (48.9)	41,207 (48.7)	<.0001
**Female**	186,706 (50.0)	23,754 (48.4)	26,494 (48.5)	28,678 (49.4)	30,388 (50.1)	34,009 (51.1)	43,383 (51.3)	
**Age (years), Mean (SD)**	68.94 (15.32)	67.25 (15.35)	68.18 (15.3)	68.74 (15.2)	69.28 (15.09)	69.62 (15.26)	69.76 (15.38)	<.0001
**18 ≤ age < 30**	8184 (2.2)	1265 (2.6)	1241 (2.3)	1198 (2.1)	1199 (2.0)	1419 (2.1)	1862 (2.2)	<.0001
**30 ≤ age < 40**	11,380 (3.0)	1824 (3.7)	1748 (3.2)	1752 (3.0)	1740 (2.9)	1867 (2.8)	2449 (2.9)	
**40 ≤ age < 50**	23,446 (6.3)	3518 (7.2)	3733 (6.8)	3809 (6.6)	3616 (6.0)	3857 (5.8)	4913 (5.8)	
**50 ≤ age < 60**	48,803 (13.1)	6968 (14.2)	7377 (13.5)	7741 (13.3)	8017 (13.2)	8358 (12.6)	10,342 (12.2)	
**60 ≤ age < 70**	66,930 (17.9)	9579 (19.5)	9932 (18.2)	10,186 (17.5)	10,497 (17.3)	11,664 (17.5)	15,072 (17.8)	
**70 ≤ age < 80**	115,087 (30.8)	15,112 (30.8)	17,345 (31.8)	18,592 (32.0)	19,192 (31.7)	20,188 (30.4)	24,658 (29.2)	
**80 ≤ age**	99,709 (26.7)	10,850 (22.1)	13,246 (24.3)	14,814 (25.5)	16,347 (27.0)	19,158 (28.8)	25,294 (29.9)	
**Comorbidities,** ***n*** **(%)**								
**Hypertension**	174,364 (46.7)	22,644 (46.1)	25,817 (47.3)	27,932 (48.1)	29,272 (48.3)	31,697 (47.7)	37,002 (43.7)	<.0001
**Diabetes**	88,157 (23.6)	11,548 (23.5)	13,232 (24.2)	14,352 (24.7)	15,101 (24.9)	16,122 (24.2)	17,802 (21.0)	<.0001
**Liver cirrhosis**	27,662 (7.4)	3963 (8.1)	4246 (7.8)	4401 (7.6)	4612 (7.6)	4751 (7.1)	5689 (6.7)	<.0001
**Chronic kidney disease**	51,157 (13.7)	6453 (13.1)	7271 (13.3)	8087 (13.9)	8563 (14.1)	9431 (14.2)	11,352 (13.4)	<.0001
**Malignancy**	114,562 (30.7)	15,387 (31.3)	17,228 (31.5)	17,999 (31.0)	18,862 (31.1)	20,083 (31.2)	25,003 (29.6)	<.0001
**locoregional solid tumor**	101,320 (27.1)	13,735 (28.0)	15,011 (27.5)	15,809 (27.2)	16,688 (27.5)	17,801 (26.8)	22,276 (26.3)	<.0001
**metastasis**	18,938 (5.1)	2844 (5.8)	2933 (5.4)	3044 (5.2)	3159 (5.2)	3240 (4.9)	3718 (4.4)	<.0001
**hematological malignancies**	16,911 (4.5)	2105 (4.3)	2822 (5.2)	2793 (4.8)	2783 (4.6)	2947 (4.4)	3461 (4.1)	<.0001
**Elixhauser comorbidity index, Mean (SD)**	18.58 (12.82)	18.81 (12.87)	18.88 (12.84)	18.69 (12.91)	18.60 (12.92)	18.53 (12.74)	18.20 (12.72)	<.0001
**index < 10**	101,136 (27.1)	13,059 (26.6)	14,332 (26.2)	15,632 (26.9)	16,522 (27.3)	17,990 (27.0)	23,601 (27.9)	<.0001
**10 ≤ index < 20**	106,916 (28.6)	14,107 (28.7)	15,518 (28.4)	16,569 (28.5)	17,104 (28.2)	19,076 (28.7)	24,542 (29.0)	
**20 ≤ index < 30**	88,271 (23.6)	11,481 (23.4)	13,064 (23.9)	13,749 (23.7)	14,365 (23.7)	15,819 (23.8)	19,793 (23.4)	
**30 ≤ index**	77,216 (20.7)	10,469 (21.3)	11,708 (21.4)	12,142 (20.9)	12,617 (20.8)	13,626 (20.5)	16,654 (19.7)	
**Insurance state,** ***n*(%)**								
**National health insurance**	323,252 (86.5)	42,251 (86.0)	47,202 (86.4)	50,264 (86.5)	52,615 (86.8)	57,737 (86.8)	73,183 (86.5)	0.001
**Medical aids**	50,287 (13.5)	6865 (14.0)	7420 (13.6)	7828 (13.5)	7993 (13.2)	8774 (13.2)	11,407 (13.5)	
**ICU admission,** ***n*(%)**	64,023 (17.1)	9681 (19.7)	10,416 (19.1)	10,529 (18.1)	10,386 (17.1)	10,446 (15.7)	12,565 (14.9)	<.0001
**Mechanical ventilation,** ***n*(%)**	103,531 (27.7)	16,099 (32.8)	17,805 (32.6)	17,633 (30.4)	16,988 (28.0)	16,643 (25.0)	18,363 (21.7)	< 0.001
**Continuous renal replacement therapy,** ***n*(%)**	64,023 (17.1)	2707 (5.5)	3237 (5.9)	3778 (6.5)	4082 (6.7)	4571 (6.9)	5168 (6.1)	< 0.001
**Type of hospital,** ***n*(%)**								< 0.001
**Tertiary hospital**	147,802 (39.6)	20,284 (41.3)	21,776 (39.9)	23,154 (39.9)	23,388 (38.6)	25,597 (38.5)	33,603 (39.7)	
**General hospital**	225,737 (60.4)	28,832 (58.7)	32,846 (60.1)	34,938 (60.1)	37,220 (61.4)	40,914 (61.5)	50,987 (60.3)	

Numbers reported as *n* (%), mean (standard deviation)

*Abbreviations*: *ICU*, intensive care unit, SD, standard deviation

### Prevalence of five comorbidities in patients with sepsis

**Fig. 1:**
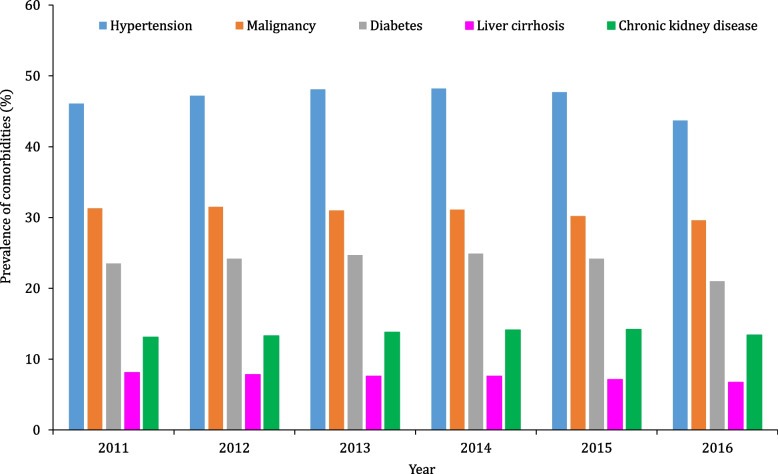
Prevalence of comorbidities from 2011 to 2016

Of the five comorbidities, hypertension was the most common at 46.7%, followed by cancer at 30.7% in patients with sepsis. The prevalence trend of these comorbid conditions remained unchanged from 2011 to 2016, with hypertension being the most common, followed by cancer among the five comorbidities in patients with sepsis (Fig. 1).

### Incidence of sepsis

**Fig. 2:**
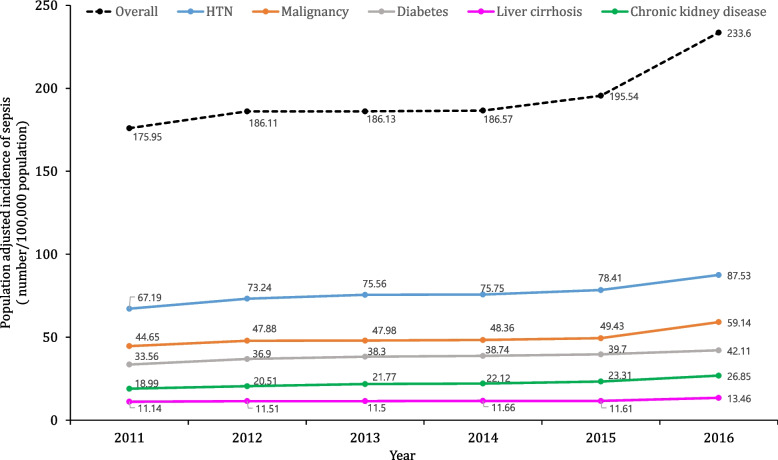
Trends of incidence of sepsis adjusting age and sex

**Fig. 3:**
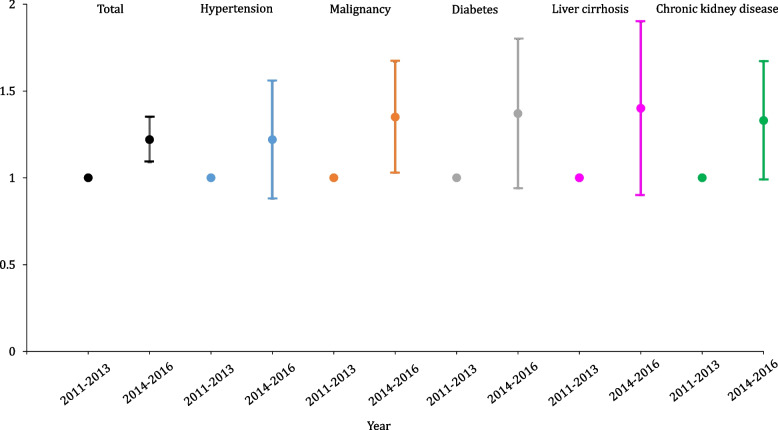
Incidence rate ratio of sepsis in relation to comorbidities and period

The annual incidence rate of sepsis per 100,000 population, adjusted for sex and age, has increased over time, from 175.95 in 2011 to 233.6 in 2016 (Fig. 2). The same pattern is seen when patients with sepsis are categorized by the five comorbidities, with the incidence rate per 100,000 population increasing over the years. The incidence rate ratio of sepsis adjusted for sex, age, and the mean Elixhauser comorbidity index showed that liver cirrhosis had the highest incidence rate ratio among the five comorbidities (Fig. 3). In other words, the incidence rate of sepsis in patients with liver cirrhosis increased by approximately 1.4 times from 2011 to 2013 to 2014–2016, which was the largest increase.

### In-hospital mortality

**Fig. 4:**
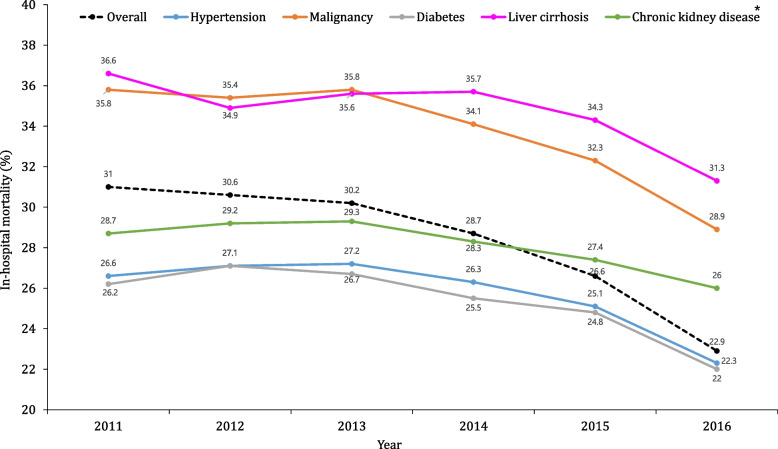
Trends of in-hospital mortality according to comorbidities. ^*^in-hospital mortality of sepsis with chronic kidney disease did not significantly decreased form 2011 to 2016 although the incidence of sepsis significantly increased over time

From 2011 to 2016, in-hospital mortality of patients with sepsis significantly decreased from 31 to 22.9% while the incidence of sepsis increased over time (Fig. 4). When analyzed by comorbid conditions, a similar trend of decreasing mortality over time was observed. Sepsis patients with metastatic cancer had the highest in-hospital mortality rate (38.4%), followed by patients with liver cirrhosis (34.5%). And liver cirrhosis, cancer, chronic kidney disease, age equal to or greater than 60, male patients, Elixhauser comorbidity index equal to or greater than 10, use of MV, and use of CRRT were associated with in-hospital mortality in patients with sepsis (Table 2).

**Table 2:** Univariable and multivariable logistic analyzes for in-hospital mortality in patients with sepsis

	In-hospital mortality	Unadjusted		Adjusted	
	*N* = 103,931	OR (95% CI)	*P*	OR (95%CI)	*P*
**Age (years),** ***n*(%)**					
**18 ≤ age < 60**	19,359 (21.1)	Reference		Reference	
**60 ≤ age < 70**	17,215 (26.2)	1.326 (1.295–1.357)	< 0.001	1.402 (1.365–1.441)	< 0.001
**70 ≤ age < 80**	33,048 (28.7)	1.507 (1.477–1.538)	< 0.001	1.910 (1.864–1.957)	< 0.001
**80 ≤ age**	34,012 (34.1)	1.937 (1.898–1.977)	< 0.001	3.233 (3.153–3.315)	< 0.001
**Sex,** ***n*(%)**					
**Male**	58,685 (31.4)	Reference		Reference	
**Female**	45,246 (24.2)	0.698 (0.688–0.709)	< 0.001	0.842 (0.828–0.857)	< 0.001
**Comorbidities,** ***n*(%)**					
**Hypertension**	55,427 (25.5)	0.807 (0.795–0.819)	< 0.001	0.627 (0.615–0.639)	< 0.001
**Diabetes**	22,206 (25.2)	0.839 (0.825–0.854)	< 0.001	0.873 (0.854–0.891)	< 0.001
**Liver cirrhosis**	9550 (34.5)	1.405 (1.369–1.442)	< 0.001	1.613 (1.564–1.664)	< 0.001
**Chronic kidney disease**	14,305 (28.0)	1.008 (0.987–1.029)	0.448	1.067 (1.040–1.096)	< 0.001
**Malignancy**	38,196 (33.3)	1.470 (1.448–1.458)	< 0.001		
**locoregional solid tumor**	33,717 (33.3)	1.435 (1.413–1.458)	< 0.001	1.832 (1.794–1.870)	< 0.001
**metastasis**	7277 (38.4)	1.666 (1.616–1.717)	< 0.001	2.218 (2.050–2.208)	< 0.001
**hematological malignancies**	5650 (33.4)	1.319 (1.276–1.363)	< 0.001	2.095 (2.014–2.179)	< 0.001
**Elixhauser comorbidity index**	NA				
**index < 10**		Reference		Reference	
**10 ≤ index < 20**		1.393 (1.365–1.421)	< 0.001	1.156 (1.127–1.183)	< 0.001
**20 ≤ index < 30**		1.552 (1.521–1.584)	< 0.001	1.161 (1.121–1.191)	< 0.001
**30 ≤ index**		1.466 (1.435–1.498)	< 0.001	0.991 (0.963–1.020)	0.539
**Insurance state,** ***n*(%)**					
**National health insurance**	89,052 (27.5)	Reference		Reference	
**Medical aid**	14,879 (29.6)	1.105 (1.083–1.128)	< 0.001	1.205 (1.177–1.235)	< 0.001
**ICU admission,** ***n*(%)**	25,564 (39.9)	1.961 (1.927–1.996)	< 0.001	0.877 (0.852–0.903)	< 0.001
**Mechanical ventilation,** ***n*(%)**	NA	7.425 (7.306–7.545)	< 0.001	8.127 (7.969–8.288)	< 0.001
**Continuous renal replacement therapy**	15,910 (67.6)	6.203 (6.030–6.382)	< 0.001	3.152 (3.048–3.259)	< 0.001
**Type of hospital,** ***n*(%)**					
**Tertiary hospitals**	39,875 (27.0)	Reference		Reference	
**General hospitals**	64,056 (28.4)	1.092 (1.076–1.108)	< 0.001	1.390 (1.359–1.422)	< 0.001

Numbers reported as *n* (%), mean (standard deviation)

Abbreviations: *CI* confidential interval, *ICU* intensive care unit, *OR* odds ratio, *SD* standard deviation

### ICU length of stay and hospital length of stay for patients admitted to the ICU

**Fig. 5:**
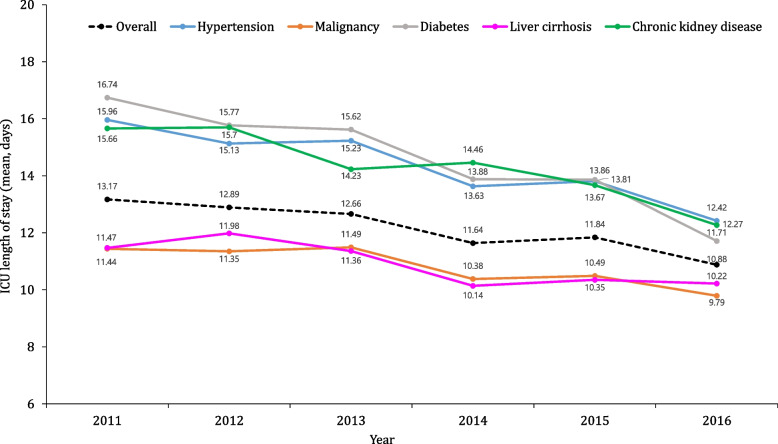
Trends of ICU length of stay based on comorbidities. ICU, intensive care unit

Out of 373,539 patients with sepsis, 17.1% (64,023) were admitted to the ICU. The average ICU length of stay for these patients was 12.1 ± 21.3 days, while the average hospital stay was 34.7 ± 45.0 days. Among the five comorbid conditions, patients with sepsis and diabetes had the longest average ICU stay at 15 days, while patients with sepsis and liver cirrhosis had the shortest average ICU stay at 11 days (Fig. 5). The average hospital stay was the longest for patients with diabetes or hypertension at 30 days, while liver cirrhosis patients had the shortest average hospital stay.

## Discussion

In this study, we investigated prevalence of five chronic medical comorbid conditions including hypertension, DM, CKD, and malignancy, and the impact of comorbidities on the outcomes of patients with sepsis in South Korea. We found that hypertension was the most prevalent comorbidity in about 47% of patients with sepsis. Among five comorbidities, LC, CKD, and malignancy were independent significant risk factors of in-hospital mortality in patients with sepsis.

A previous study demonstrated that the annual incidence of sepsis increased, whereas in-hospital mortality decreased from 2011 to 2015 in South Korea [14]. Similarly, in our study, the prevalence of the five comorbidities and their incidence rate ratio in patients with sepsis increased over time; however, the in-hospital mortality rate of patients with these comorbidities and sepsis decreased between 2011 and 2016. The improved survival over time may be attributed to advancements in supportive care and prompt and protocol-driven treatment in accordance with international guidelines [13, 15], including protective MV, early effective antibiotic treatment, and rapid fluid resuscitation. Although prognosis of sepsis has improved, the majority of underlying causes of death were still associated with severe chronic comorbidities and the majority of sepsis-related deaths [16].

### Hypertension

The worldwide prevalence of hypertension was estimated at 1.13 billion in 2015 [17], and increased with age, with 53.3% prevalent in people 50 years of age [18]. Similarly, approximately half the patients with sepsis had hypertension as one of the comorbidities in this study. However, there is a paucity in published studies on how hypertension affects clinical outcome in patients with sepsis. We found that hypertension had lower in-hospital mortality than other comorbidities, in line with previous ICU studies [19, 20]. A retrospective study demonstrated that patients with septic shock and chronic hypertension achieved and maintained target mean arterial pressure (MAP) more quickly, but that faster target MAP attainment and maintenance did not translate into lower mortality [21]. Therefore, further study is needed to investigate the impact of hypertension on clinical outcomes and the related mechanisms.

### Diabetes mellitus (DM)

The prevalence of diabetes in patients with sepsis was more than twice as higher as 10.8% in adults aged 30 and older in South Korea in 2016 [22]. Although patients with DM have an increased susceptibility to develop sepsis due to alterations in the host response [23, 24], the impact on ICU outcomes varies [25]. In this study, in-hospital mortality in diabetic patients with sepsis showed the lowest mortality among five comorbidities. Similarly, a previous study showed that diabetic patients with sepsis had lower mortality rates than nondiabetic patients with sepsis [26]. Previous studies have shown an association between abnormal glucose levels and mortality in patients with sepsis [27, 28]. However, the effect of glucose variability on mortality was not replicated in diabetic patients with sepsis, suggesting a protective effect of DM [29].

### Liver cirrhosis (LC)

LC has been one of the co-morbidities associated with the highest mortality rate in patients with sepsis. Our study shows a 7.4% prevalence of liver cirrhosis (LC), notably higher than the 0.3% to 0.8% seen in European population-based studies [30]. This can be partly attributed to South Korea’s high hepatitis B virus (HBV) carriage rates (around 10% until the early 1990s) [31] and the rise in alcohol-related LC post-2008 [32]. Additionally, the fact that about 78% of South Korean patients receive care in tertiary and general hospitals may also influence these results. This prevalence is comparable to a French multicenter study [33], which found a 7.6% prevalence of LC in septic shock patients, similar to our findings. In this study, compared to 2011 ~ 2013, the incidence rate of sepsis in patients with cirrhosis was increased 1.4 times during 2014 ~ 2016; however, in-hospital mortality decreased by 12.1% from 2011 to 2016. These results are consistent with those of previous ICU studies [33, 34]. Similarly, as seen in sepsis patients with other comorbidities, a combination of factors, including sepsis management, attention to the prevention of hospital-acquired conditions, and the organization of the ICU has increased survival to discharge in patients with non-hepatic reasons for admission [35].

### Chronic kidney disease (CKD)

Chronic kidney disease is a common comorbidity. Its prevalence exceeds 13% in the US [36] and 8.2% in Korea adult population [37]. Moreover, observational studies reported a 10% to 13.3% prevalence in ICU patients [19, 38], similar to the 13.7% in our patients with sepsis. The majority of studies reported variable in-hospital mortality rates for patients with CKD ranging from 28% to 45% [9, 10], which is in line with our results, at 28%. Uremia in these patients is associated with increasing the risk of bacterial infections through several mechanisms, including impaired neutrophil activation and cellular immunity [39, 40]. In addition, the relation with other organ dysfunction and the role in AKI development made CKD contribute the significant risk of in-hospital mortality in patients with sepsis. Furthermore, CKD is present in approximately 30% of ICU patients with acute kidney injury (AKI) [41] and is a significant risk factor for developing AKI [42]. The relationship with other organ dysfunction and its role in AKI development makes CKD a significant risk factor for in-hospital mortality in patients with sepsis.

### Malignancy

Malignancy is the second most common comorbidity in this population. And it is also one of the most common comorbid conditions at approximately 17% of sepsis patients in the United States [2]. Despite the increased incidence of sepsis in patients with malignancy, several factors, including advances in the treatment of malignancy and earlier ICU admission, may explain the decreasing trend in in-hospital mortality, as shown in our study [43]. The mortality rates in this study are lower than those reported in previous studies [44, 45]. This difference can be explained by a higher proportion of locoregional solid tumors in sepsis patients with malignancy, and lower severity representing 40% of ICU admission of all sepsis patients than those in previous studies. Notably, the presence of organ dysfunction was associated with increased mortality, whereas the presence of neutropenia and disease progression were not, suggesting that the risk of death in these patients was related to the intensity of sepsis rather than to the characteristics of the underlying malignancy [44].

### Other risk factors of in-hospital mortality

Except for the three comorbidities, MV, CRRT, patients covered under medical aid, and patients admitted in general hospitals were significant risk factors of in-hospital mortality in our study. Organ dysfunction is one of the defining factors of sepsis [46] and is associated with a poor prognosis for patients with sepsis [47]. MV and CRRT are supportive therapies for organ dysfunction. We found that patients on MV had an eight-fold higher in-hospital mortality risk than those not receiving MV. In addition, patients undergoing CRRT had a four-fold higher risk of death than those not receiving CRRT in this study. Approximately 13% of patients with sepsis in present study’s population are covered by the Medical Aid Program, which is much higher than the 3% of the general population covered by the Medical Aid Program in Korea. Previous studies demonstrated a dose-dependent association between low income and the prevalence of medical comorbidities and all-cause mortality [48, 49].

The main strengths of this study were the large sample size and the heterogeneity of the Korean inpatient population across all general and tertiary hospitals over 6 years, which made it representative of the entire country. And this is the first population-based study in Korea to investigate prevalence of several comorbidities and its impact on in-hospital mortality in patients with sepsis.

### Limitations

There are also several limitations to consider. First, we used the Sepsis-2 definition for selection of patients with sepsis, as the Sepsis-3 definition requires sepsis-related organ dysfunction. Using the Sepsis-3 definition, the incidence of sepsis and related mortality may differ. However, there was an overlap of 92%, according to a study that compared the two definitions in identifying patients with sepsis [50]. Although the study was conducted in ICU patients, it can be inferred that identification of sepsis according to the Sepsis-2 and Sepsis-3 definitions is highly concordant. The definition of the study population took into account the limitations of the database in identification of sepsis-associated organ dysfunction and the consistency of identification of patients with sepsis over the study period. Second, the database used in our study was designed for reimbursement purposes and consisted of codes for disease, procedures, medications and specific outcomes such as mortality and LOS. But, there was a lack of laboratory and physiological data. Additionally, the database did not link time data to disease codes; hence, it was not possible to distinguish whether the primary diagnosis was sepsis on admission or sepsis that developed during the hospitalization. Nevertheless, considering the inclusion of almost all sepsis episodes in Korea from 2011 to 2016, it is clear from the results of this study that the incidence of sepsis is increasing, but the associated hospital mortality is decreasing. Third, assessment of the severity of illness at admission and the cause of sepsis, both of which could affect in-hospital mortality, were missing from the database. The proportion of patients requiring ICU admission may reflect severity of illness, but it would be very difficult to distinguish whether sepsis or a complication of sepsis was the cause of ICU admission. The use of the Elixhauser Comorbidity Index is the most widely accepted method of risk adjustment in the analysis of administrative healthcare data and was therefore the method of choice for this study. Fourth, unfortunately, our research did not include the collection of vasopressor usage data. We evaluated sepsis severity using alternative indicators, namely ICU admissions and forms of organ support such as mechanical ventilation and Continuous Renal Replacement Therapy (CRRT). It is important to note that Oh et al. [14] identified factors such as male sex, older age, and a higher Elixhauser comorbidity index, along with mechanical ventilation use, as significant for in-hospital mortality, but did not explicitly address vasopressor use. This influenced our methodology, leading us to focus on these specific severity indicators in our study. Finally, we analyzed the impact of separate comorbidities on outcomes in patients with sepsis after adjusting for the Elixhauser Comorbidity Index. Nonetheless, it is difficult to evaluate the impact of the number of comorbidities or the combination of comorbidities on outcomes in these populations.

In conclusion, hypertension was the most prevalent comorbidity in patients with sepsis. The incidence of sepsis in patients with hypertension, DM, LC, CKD, or malignancy increased with decreasing trends in the in-hospital mortality rate from 2011 to 2016 in South Korea. Additionally, LC, CKD, and malignancy result in higher in-hospital mortality rates than hypertension and DM and are significant risk factors for in-hospital mortality in patients with sepsis. Further studies integrating laboratory and physiological data are needed to determine the impact of comorbidities and related organ dysfunction in patients with sepsis.

### Supplementary Information

**Additional file 1: Supplemental Table 1.** ICD-10 codes associated with sepsis.

## Data Availability

The datasets used and/or analyzed during the current study are available from the corresponding author on reasonable request.
