# Supplementary material for: Prevalence and outcomes of chronic comorbid conditions in patients with sepsis in Korea: a nationwide cohort study from 2011 to 2016
Source: BMC Infect Dis. 2024 Feb 12;24:184. doi: 10.1186/s12879-024-09081-x (PMC10860243; doi:10.1186/s12879-024-09081-x)
Supplement: Supplementary file 1 — Additional file 1: Supplemental Table 1. ICD-10 codes associated with sepsis. [file 12879_2024_9081_MOESM1_ESM.docx]

**Supplemental Table 1. ICD-10 codes associated with sepsis**

| **ICD-10 version 2016** | **Diseases** |
| --- | --- |
| A02.1 | Salmonella sepsis |
| A04.7 | Enterocolitis due to Clostridium difficile |
| A20.7 | Septicemic plague |
| A22.7 | Anthrax sepsis |
| A26.7 | Erysipelothrix sepsis |
| A32.7 | Listerial sepsis |
| A39.4 | Meningococcemia, unspecified |
| A40 | Streptococcal sepsis |
| A40.0 | Sepsis due to streptococcus, group A |
| A40.1 | Sepsis due to streptococcus, group B |
| A40.2 | Sepsis due to streptococcus, group D |
| A40.3 | Sepsis due to Streptococcus pneumoniae |
| A40.8 | Other streptococcal sepsis |
| A40.9 | Streptococcal sepsis, unspecified |
| A41 | Other sepsis |
| A41.0 | Sepsis due to Staphylococcus aureus |
| A41.1 | Sepsis due to other specified staphylococcus |
| A41.2 | Sepsis due to unspecified staphylococcus |
| A41.3 | Sepsis due to Hemophilus influenza |
| A41.4 | Sepsis due to anaerobes |
| A41.5 | Sepsis due to other Gram-negative organisms |
| A41.8 | Other specified sepsis |
| A41.9 | Sepsis, unspecified |
| A42.7 | Actinomycotic sepsis |
| A49.9 | Bacterial infection, unspecified |
| B37.7 | Candidal sepsis |
| B9548 | Streptococcal necrotizing enterocolitis |
| B956 | Staphylococcus aureus as the cause of diseases classified to other chapters |
| B962 | Escherichia coli [E. coli] as the cause of diseases classified to other chapters |
| O85 | Puerperal sepsis |
| R57.2 | Septic shock |
| R65.0 | Systemic Inflammatory Response Syndrome of infectious origin without organ failure |
| R65.1 | Systemic Inflammatory Response Syndrome of infectious origin with organ failure |
